# Synergic Effect of Dendrite‐Free and Zinc Gating in Lignin‐Containing Cellulose Nanofibers‐MXene Layer Enabling Long‐Cycle‐Life Zinc Metal Batteries

**DOI:** 10.1002/advs.202202380

**Published:** 2022-07-07

**Authors:** Chaozheng Liu, Zhenglin Li, Xiaoman Zhang, Wangwang Xu, Weimin Chen, Kangning Zhao, Yao Wang, Shu Hong, Qinglin Wu, Mei‐Chun Li, Changtong Mei

**Affiliations:** ^1^ Co‐Innovation Center of Efficient Processing and Utilization of Forest Resources College of Materials Science and Engineering Nanjing Forestry University Nanjing 210000 P. R. China; ^2^ Mechanical & Industrial Engineering Department Louisiana State University Baton Rouge LA 70803 USA; ^3^ College of Materials and Chemical Engineering China Three Gorges University Yichang 443002 P. R. China; ^4^ Laboratory of Advanced Separations (LAS) École Polytechnique Fédérale de Lausanne (EPFL) Sion CH‐1950 Switzerland; ^5^ Hollingsworth & Vose (Suzhou) Co. Ltd Suzhou Industrial Park Suzhou 215126 P. R. China; ^6^ School of Renewable Natural Resources Louisiana State University Agricultural Center Baton Rouge LA 70803 USA

**Keywords:** diffusion limited dendrite suppression, lignin‐containing cellulose nanofibers, MXene, zinc gate, zinc ion batteries

## Abstract

Uncontrollable zinc dendrite growth and parasitic reactions have greatly hindered the development of high energy and long life rechargeable aqueous zinc‐ion batteries. Herein, the synergic effect of a bifunctional lignin‐containing cellulose nanofiber (LCNF)‐MXene (LM) layer to stabilize the interface of zinc anode is reported. On one hand, the LCNF provides enough strength (43.7 MPa) at relative low porosity (52.2%) to enable the diffusion limited dendrite suppression, while, on the other hand, the MXene serves as a zinc gating layer, facilitating the zinc ion mobility, restricting the active water/anions from degradation in the electrode/electrolyte interface, and epitaxially guiding zinc deposition along (002) plane. Benefiting from the synergic effect of diffusion limited dendrite suppression and zinc gate, the LM layer enabled a high coulombic efficiency (CE) of 98.9% with a low overpotential of 43.1 mV at 1 mA cm^−2^ in Zn//Cu asymmetric cells. More importantly, Zn//MnO_2_ full cells with the LM layer achieve a high‐capacity retention of 90.0% for over 1000 cycles at 1 A g^−1^, much higher than the full cell without the protective layer (73.9% over 500 cycles). The work provides a new insight in designing a dendrite‐free zinc anode for long‐cycle‐life batteries.

## Graphical abstract

The synergic effect of mechanical induced dendrite suppression and zinc gating layer to enable corrosion‐free zinc anode for long‐cycle‐life batteries with the lignin‐containing cellulose nanofiber (LCNF)‐MXene layer is implemented, in which LCNFs act as a desolvation layer to suppress water‐induced corrosion reactions while the MXene serves as a zinc gating layer to guide dendrite formation in the lateral direction.

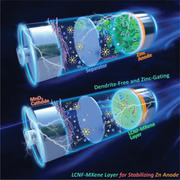

## 1 Introduction

Aqueous rechargeable batteries applied as reliable energy storage devices have received considerable attention due to their intrinsic safety and low cost.^[^ ^1^ ^]^ Among various candidates, rechargeable aqueous zinc‐ion batteries (RAZIBs) are one of the best choices due to the attractive features of Zn metal anode, including its abundant resources, cost effectiveness, low redox potential (−0.76 V vs standard hydrogen electrode), and high theoretical capacity (820 mAh g^−1^ and 5855 mAh cm^−3^).^[^ ^2^ ^]^ However, the practical application of RAZIBs is impeded by the unstable Zn anode/electrolyte interface caused by the uncontrollable Zn dendrite growth and parasitic reactions. The uncontrolled formation of Zn dendrite during cycling would produce porous Zn, leading to the internal short‐circuit and the acceleration of corrosion.^[^ ^3^, ^4^ ^]^ In addition, the competing reaction between active free water‐induced hydrogen evolution reaction (HER) and the zinc deposition potential, would occupy the active sites in the zinc metal surface, greatly increasing the local current density for zinc deposition and resulting in low deposition efficiency of the RAZIBs.^[^ ^5^ ^]^ Thus, it is crucial to address both of the two challenges simultaneously to enable dendrite‐free and long‐cycle‐life zinc metal battery.

Recently, various strategies have been developed to suppress/block Zn dendrite growth and parasitic reactions, such as constructing hierarchical host structures,^[^ ^6^ ^]^ optimizing electrolytes,^[^ ^7^ ^]^ and modifying interfacial engineering.^[^ ^8^, ^9^ ^]^ As one of the most effective methods for achieving a highly‐reversible Zn anode, employing a protective interface layer with good electrochemical and mechanical properties have been investigated.^[^ ^10^ ^]^ Niu's group developed a self‐assembled MXene layer on the zinc anode to uniformize the surface electric field and smooth the dendrite‐free surface, which can effectively suppress the dendrite formation on the anode.^[^ ^11^ ^]^ To provide a further understanding of dendrite growth in mechanical point of view, the strength of the layer is crucial to the mechanical suppression of the dendrite development. Compared to lithium metal, Zn metal has a greatly higher yield strength (27 MPa) and Young's modulus (108 GPa), which could tolerate larger internal stress leading to a larger mechanical suppression effect before undergoing plastic deformation.^[^ ^12^ ^]^ In this sense, it would be easier for zinc dendrites to surpass through the separators, which is the main reason for the failure of the battery during repeated cycles compared with lithium metal.^[^ ^13^ ^]^ More importantly, this value is almost impossible to be achieved in the polymer or polymer‐based membrane to realize the dendrite suppression. Alternatively, it is predicted that finely tuning the porosity would effectively shift from the mechanical limited dendrite suppression to the diffusion limited dendrite suppression.^[^ ^14^ ^]^ Therefore, balancing the porosity and mechanical strength of the membrane would be beneficial in suppressing dendrite formation in zinc ion battery, but remained unexplored.

On the other hand, water/oxygen‐induced side reactions accompanying the zinc deposition/stripping process pose another problem in degrading the cycling performance of zinc‐ion batteries (ZIBs). Compared with the traditional aqueous alkaline electrolytes, the current neutral electrolyte had provided a greater thermodynamic stability against HERs. However, the competition reactions between HER and zinc deposition would result in less active sites for zinc deposition, leading to greatly induced areal current density in the plating/stripping area, low coulombic efficiencies, and overwhelming evolution of gas in the cell.^[^ ^15^ ^]^ These issues largely involved the active free water in the electrolyte. Therefore, restricting the free water in the interface of electrode and electrolyte would be beneficial in getting rid of the free water‐induced side reaction. Qian et al. reported that the cellulose nanowhisker‐graphene membrane was constructed to stabilize the Zn metal anode.^[^ ^16^ ^]^ This layer can not only act as a desolvation layer to hinder the dendrite reaction, but also generate a deanionization shock to shield anions for the uniform deposition of Zn^2+^. Therefore, in order to realize the property of both zinc dendrite suppression and active water restriction, an ideal protective layer would need to combine both merits of hydrophobicity that can fix the free water in between aqueous electrolyte and Zn anode as well as low porosity and enough mechanical strength to suppress the dendrite formation.

Herein, we proposed a novel lignin‐containing cellulose nanofiber (LCNF)‐MXene (LM) protective layer as an ideal interfacial layer to stabilize Zn metal anode. This LM layer with superior mechanical properties (43.7 MPa) has a dual function, in which the LCNF acts as a desolvation layer to restrict the free water molecules from the interface of electrode/electrolyte to suppress water‐induced corrosion reactions while the MXene serves as a zinc gating layer to guide dendrite formation in the lateral direction. In this way, benefiting from the synergic effect of dendrite suppression and active water/anion restricting effects, the protective layer guides the zinc crystal to grow along (002)_Zn_ direction during deposition, parallel with zinc anode surface. With this unique LM protective layer, the zinc symmetric cells and the Zn//Cu asymmetric cells deliver ultra‐long cycle life with high stabilities, indicating significantly improved reversibility of zinc anodes. Moreover, the full cell with the LM protective layer delivered a high capacity of 175.4 mAh g^−1^ with 90.0% capacity retention after 1000 cycles, demonstrating the significant potential for grid‐scale application.

## 2 Results and Discussion

As shown in Figure S1, Supporting Information, the free‐standing LM film was fabricated through vacuum filtration of a mixed suspension of LCNF and MXene, followed by air drying. For comparison, the pure LCNF film and the pure MXene film were also prepared. The rheological properties and zeta potentials of three film‐forming suspensions were studied as shown in Figure S2, Supporting Information. The LM film‐forming suspension exhibits higher viscosity and lower zeta potential compared to those of pure MXene suspension, suggesting the physical entanglement between MXene nanosheets and LCNFs.^[^ ^17^ ^]^ Additionally, the average zeta potential value of the LM film was −18.1 mV, demonstrating its negatively charged surface, which is attractive for the cations. The chemical interaction between MXene and LCNF was further investigated with X‐ray diffraction spectrometer (XRD) patterns, and the Fourier transform infrared spectrometer (FTIR) spectrum. As the results shown in Figure S3, Supporting Information (detailed analysis in the Supporting Information), LCNFs and MXene nanosheets are firmly connected with each other due to the extensive interactions of hydrogen bonds and physical entanglement.^[^ ^18^ ^]^ This was further confirmed with Scanning Electron Microscope (SEM) and Transmission electron microscopy (TEM) images in Figures S4 and S5, Supporting Information. LCNFs are firmly attached on the surface of MXene nanosheets to form an integrated laminate structure, and the MXene nanosheets are wrapped in a highly entangled network of LCNFs. Cross‐sectional SEM images (Figure S5d, Supporting Information) also revealed that the thickness of the LM film with a multilayer structure was approximately 48 µm. Benefiting from the firm connection between MXene and LCNF, the obtained LM film is believed to be endowed with remarkable mechanical properties. As depicted in Figure S6, Supporting Information, the LM film exhibits the maximum stress (43.7 MPa) with the maximum strain (6.14%), much higher than MXene film (stress: 16.0 MPa, strain: 1.1%) and LCNF film (stress: 38.6 MPa, strain: 4.6%). Thus, the LM film can act as a physical barrier to suppress zinc dendrite due to its excellent mechanical properties. Moreover, according to the nitrogen sorption measurements (Figure S7, Supporting Information), the LM film exhibits a lower specific area (3.9 m^2^ g^−1^) and a larger average pore diameter (11.6 nm) than those of the MXene film (28.0 m^2^ g^−1^ and 9.3 nm), respectively. It is believed that the lower porosity (52.2%) accompanied by the higher mechanical strength of the LM layer would shift the mechanical limited dendrite suppression to the diffusion limited dendrite suppression, which enables the dendrite‐free deposition of zinc metal.^[^ ^19^ ^]^

**Figure 1:**
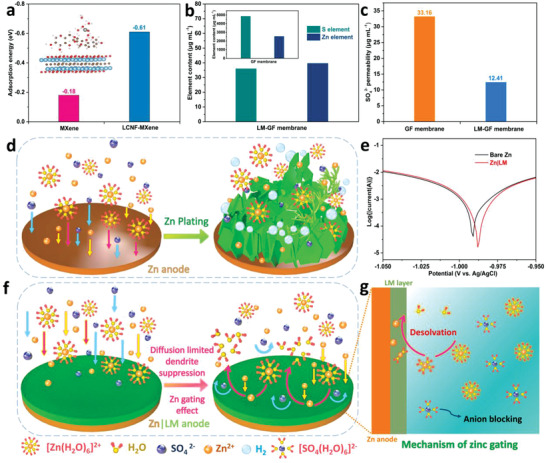
Theoretical simulation of desolvation of LM film. a) Calculated adsorption energies of hydrated Zn^2+^ for MXene and LM membrane, and the inset is the DFT simulation of desolvation effect for LM membrane. b) Zn and S elements permeability of GF and LM membrane. c) SO_4_^2−^ permeability of GF and LM membrane. d,f) Schematic illustration of desolvation of GF and LM membrane, respectively. e) LSV curves of bare Zn and Zn‐LM electrodes in 2 m ZnSO_4_ electrolyte. g) Mechanism diagram of zinc gating.

The desolvation effect was elaborated by density functional theory (DFT) simulation. As the results summarized in **Figure** **1**a, the absorption energy of hydrated zinc ion with six water molecules for MXene film was −0.18 eV, whereas for LM film, the adsorption energy was −0.61 eV, which suggests that cellulose in LM film would provide a strong interaction with H_2_O molecules to trap the active water molecule. This phenomenon is also supported by the enhanced wettability between LM layer and electrolyte when compared to pure Zn anode (Figure S8, Supporting Information), which will be beneficial to the permeability of electrolyte. When hydrated Zn^2+^ is freely transported through the LM layer to reach the Zn anode, the water shell in hydrated zinc ions would be trapped by the LM film due to the high adsorption energy as illustrated in the schematic of Figure 1d,1f. In this way, the zinc anode/electrolyte interface would be restricted by the LM layer, creating the Zn^2+^‐rich, H_2_O‐poor electrical double layer structure.^[^ ^20^ ^]^ This is also supported by the diffusion experiment. The ionic permeability of LM film was investigated by H‐cells with deionized water and 3 m ZnSO_4_ electrolyte (Figure S9, Supporting Information). After stirring for 1 h, the collected sample in deionized water was analyzed through inductively coupled plasma (ICP) and ion chromatography (IC), respectively. As the results shown in Figure 1b,c, compared with the bare glass‐fiber (GF) membrane, the proportion of S element using LM‐GF membrane decreased while the proportion of Zn element increased. Moreover, the SO_4_^2−^ permeability using LM‐GF membrane (12.41 µg mL^−1^) was much lower than that of bare GF membrane (33.16 µg mL^−1^). The selective transport of zinc ions with water/anions is expected to reduce the corrosion reaction in the zinc anode surface. Therefore, the corrosion of zinc plates is tested by linear polarization experiments. Compared with the bare Zn anode (−0.991 V, 40.921 µA), the increased corrosion potential (−0.988 V) and the reduced corrosion current (16.372 µA) of the Zn‐LM anode present the inhibition of corrosion reactions and low corrosion rate (Figure 1e). These results demonstrate that the LM layer can act as the zinc gating layer to facilitate the zinc transport through the layer while restricting active water/anions from surface of the zinc anode/electrolyte interface (Figure 1g).^[^ ^16^, ^21^ ^]^

**Figure 2:**
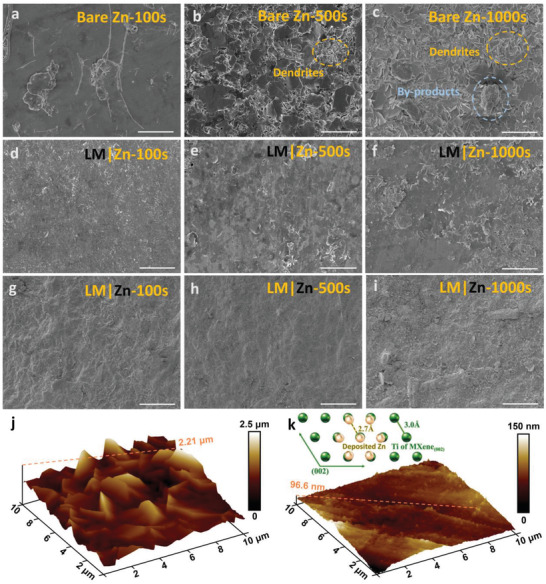
Morphology of zinc anode at a current density of 5 mA cm^−2^ after deposition for 100, 500, and 1000 s, respectively. a–i) SEM images of Bare Zn anode (a‐c), Zn anode protected by LM layer (d–f), and the LM layer (g–i) on the zinc anode surface after Zn deposition for 100, 500, and 1000 s, respectively. j,k) AFM images of Bare Zn anode and LM‐Zn anode after Zn deposition for 1000 s, and the inset is atomic arrangements of Ti‐terminated surface of MXene and deposited Zn (002) with their small lattice mismatch. Scale bar: (a–f) 10 µm; (g–i) 50 µm.

To investigate the influence of the protective layer on zinc deposition, the morphologies of deposited zinc anode with/without LM layer were observed by SEM at a current density of 5 mA cm^−2^. As shown in **Figure** **2**a–c, with the deposition time increased from 100 to 1000 s, numerous anisotropic plate‐like dendrites and corrosion byproducts appeared on bare zinc anode. This observation is consensus with the zinc plating curve, in which both the nucleation and growth potentials (77.5 and 93.1 mV, respectively) of unprotected Zn anode are larger, which reflects the rarer zinc nucleation sites as well as uncontrollable Zn dendrite growth (Figure S10, Supporting Information).^[^ ^4^ ^]^ In contrast, the protected zinc anode shows smaller nucleation and growth potentials (33.8 and 125.7 mV, respectively), suggesting the dendrite suppression mechanism. As displayed in the SEM images in Figure 2d–i, with LM layer protection, the zinc anode maintained a flat surface, indicating that the deposited Zn was paralleling with zinc anode surface and growth of dendrites was effectively modulated. Meanwhile, the morphology and elemental composition of LM layer were well maintained after Zn deposition. As the SEM image and Energydispersive X‐ray spectroscopy (EDS) mapping shown in Figure S11, Supporting Information, after Zn deposition for 1000 s, the laminated structure of LM layer was well maintained and C, Zn, and Ti elements were uniformly distributed. To further investigate the crystal structure of the deposited Zn, XRD technique was applied. As the XRD patterns shown in Figure S12, Supporting Information, the characteristic diffraction peak of LM‐Zn anode at around 36.3° corresponded to (002)_Zn_ plane gradually intensified, suggesting the preferential growth of zinc along the (002)_Zn_ plane, which is believed to be suitable for epitaxial growth of zinc in MXene surface due to the small lattice mismatch (the inset of Figure 2k).^[^ ^9^, ^22^ ^]^ Furthermore, to confirm the smooth surface, atomic force microscopy (AFM) images were collected. As shown in Figure 2j,k, after 1000 s deposition, pure zinc anode exhibits an altitude of 2.21 µm, whereas the LM‐Zn anode presents an altitude of only 96.6 nm. In addition, the in situ optical visualization observations were employed to clearly show the growth of zinc dendrites after Zn deposition. As shown in Figure S13, Supporting Information, the LM‐Zn anode exhibited a dendrite suppression effect in the whole deposition process, guiding the zinc deposition along the parallel direction, and the bare zinc anode surface presented obvious protrusions after the deposition for 1000 s. The high magnified SEM images in Figure S14, Supporting Information, confirm that the LM‐Zn anode exhibits a plate‐like morphology, which is parallel with zinc anode surface, confirming the epitaxial growth of zinc in MXene surface as well as the corrosion‐free zinc plating/stripping. These results are in consistence with the previous ex situ SEM images, XRD, and AFM results, justifying that the LM protective layer guided the oriented deposition of Zn^2+^ on the anode. Moreover, the EIS results in Figures S15 and S16, Supporting Information, demonstrate the low charge transfer conductivity (2.89 × 10^−4^ S cm^−1^) and high ionic conductivity (1.55 × 10^−2^ S cm^−1^) of the LM protective layer. Thus, it is believed that as an artificial interface layer, the LM layer with the property of diffusion limited dendrite suppression, zinc gating layer to avoid corrosion reaction, and guided epitaxial zinc deposition would be beneficial for extending the cycle life of zinc metal battery.

**Figure 3:**
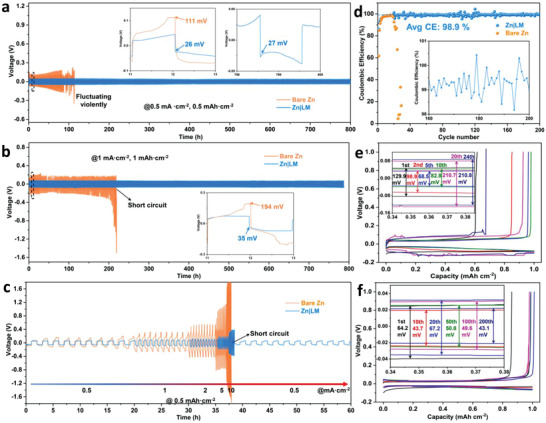
Electrochemical performance of zinc symmetric cells and Zn//Cu asymmetric cells. a–c) Galvanostatic cycling performance of zinc symmetric cells with/without the LM layer at a current density of 0.5 mA cm^−2^ with a capacity of 0.5 mAh cm^−2^ (a) and 1 mA cm^−2^ with a capacity of 1 mAh cm^−2^ (b) and rate performance at a fixed capacity of 1 mAh cm^−2^ (c), respectively. d) Coulombic efficiencies of Zn//Cu asymmetric cells with/without LM layer at 1 mA cm^−2^. e,f) Galvanostatic charge/discharge profiles of Zn//Cu asymmetric cells without or with LM layer at selected cycles in 2 m ZnSO_4_ electrolyte at 1 mA cm^−2^ with a voltage cut‐off of 1 V.

The electrochemical stability of zinc anodes with/without the LM protective layer was initially evaluated in symmetric cells under galvanostatic conditions. At a current density of 0.5 mA cm^−2^ and a limited capacity of 0.5 mAh cm^−2^ (**Figure** **3**a), the bare Zn//Zn symmetric cells exhibit a polarization voltage of 111 mV at early cycles, followed by obvious fluctuations after 40 charge‐discharge cycles (80 h), and finally abrupt failure at the 57th cycle. As a comparison, the Zn|LM//Zn|LM symmetric cells exhibit highly stable cycling performance for 800 h (400 cycles) with a low polarization voltage of 27 mV. With the current density increased to 1 mA cm^−2^ and capacity increased to 1 mAh cm^−2^, the bare Zn//Zn cell becomes short‐circuited after 110 cycles (219 h) with an initial polarization voltage of 194 mV (Figure 3b). While the symmetric cell equipped with the LM layer shows a longer lifespan (400 cycles, ≈788 h) and a much lower overpotential of 35 mV. With the current density further increased to 2 mA cm^−2^ with capacity of 2 mAh cm^−2^, the Zn|LM//Zn|LM cell operates stably after 158 cycles (more than 300 h). In contrast, short‐circuit occurs in Zn//Zn cells after only 34 cycles (65 h) (Figure S17a, Supporting Information). Even at high current densities and high area capacities, the Zn plating/stripping stability in Zn symmetric cell was improved by the LM protective layer (Figure S17b, Supporting Information). Figure 3c displays the rate performance of both Zn|LM//Zn|LM and Zn//Zn cells. All the rate profiles clearly reveal that the Zn|LM//Zn|LM cell exhibit much lower polarization voltages and a longer cycling lifespan than those of the bare Zn//Zn cell at different current density. It is well known that the polarization is caused by ohmic hindrance and slow mass transport. The significant increase of the polarization voltage of Zn symmetric cells is attributed to the uneven plating/stripping of zinc and corrosion reaction on the Zn/electrolyte interface, resulting in the slow zinc mass transport and high electron resistance,^[^ ^23^ ^]^ which is also supported by SEM observation and EIS measurement. The surface morphology of zinc plate without LM layer in Figure S18a, Supporting Information, reveals the excess growth of zinc dendrite in the vertical direction while the one with LM layer shows a pretty flat surface, suggesting the lateral direction. The EIS impedance after cycling in Figure S19, Supporting Information, reveals the greatly increased charge transfer resistance, suggesting that the excessive growth of the dendrite in vertical direction would increase the charge transfer resistance and finally lead to short‐circuit. To further illustrate the effect of LM layer on the reversibility of Zn plating/stripping, the coulombic efficiency (CE, i.e., the ratio of Zn stripping capacity to Zn plating capacity) of the Zn//Cu asymmetric cells was also investigated. As illustrated in Figure 3d,e, the CE and the galvanostatic charge‐discharge (GCD) profiles of the Zn//Cu asymmetric cells with or without an LM layer were performed at 1 mA cm^−2^ with a voltage cut‐off of 1 V, respectively. For these two cells, the initial decrease of the polarization voltage in the first few cycles is attributed to the activation and interfacial wetting process. The CE starts to decay after 18 cycles and then the cell finally fails in 30 cycles. The CE at the 24th cycle is only 67.5% with a high overpotential of 210.8 mV, suggesting the rapid growth of Zn dendrite and the formation of “dead Zn”. In contrast, the Zn|LM//Cu cell delivers an average CE of 98.9% for over 200 cycles, and its overpotential gradually decreases from 67.2 mV at the 20th cycle to 43.1 mV at the 200th cycle (Figure 3f), demonstrating the highly reversible of the Zn plating/stripping on the anode. Such results indicated the better electrochemical kinetics of the Zn//Cu asymmetric cells with LM protective layer than the pristine one.

**Figure 4:**
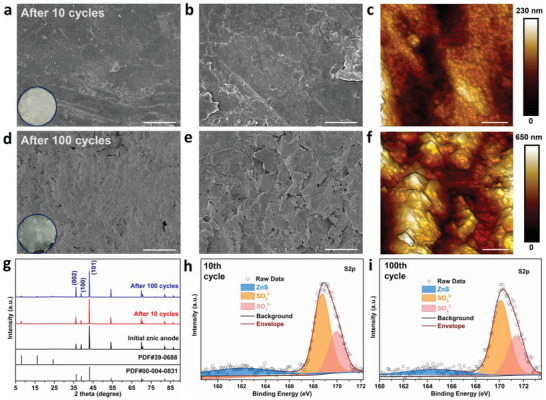
Electrochemical deposition mechanism of the zinc symmetric cells with LM layer after cycling at a current density of 1 mA cm^−2^ with a capacity of 1 mAh cm^−2^. a–f) Top‐view SEM images of LM‐Zn anodes after 10 cycles (a, b) and 100 cycles (d, e), and AFM images of LM‐Zn anodes after 10 (c) and 100 (f) cycles. g) XRD patterns of LM‐Zn anodes after cycling. h,i) XPS spectra of S 2p for LM‐Zn anodes after 10 (h) and 100 (i) cycles. Scale bar: (a, d) 100 µm; (b, e) 20 µm.

To investigate the electrochemical deposition mechanism, the surface morphology of the zinc anode in Zn|LM//Zn|LM cells after cycling at a current density of 1 mA cm^−2^ with a capacity of 1 mAh cm^−2^ was further investigated. The surface profiles of Zn|LM electrode at different cycling numbers were characterized by the ex situ SEM and AFM techniques, and the results are shown in **Figure** **4**. After 10 cycles, the Zn|LM electrode shows a quite flat and smooth surface (Figure 4a,b), with an average height difference of only 230 nm (Figure 4c). Upon 100 cycles, a dense and flat layer was exhibited on the surface of Zn|LM electrode (Figure 4d–f). In contrast, numerous rough dendrite flakes are observed on bare zinc anode, forming a loose and rough surface (Figure S18a, Supporting Information). Ex situ XRD and X‐ray photoelectron spectroscope (XPS) analyses are also carried out. As illustrated in Figure 4g, the crystal structure of the initial zinc anode before cycling is assigned to that of commercial zinc foil (PDF#00‐004‐0831). The intensity ratios of the (002) plane to the (100) plane of the zinc anode with the LM layer after cycling are calculated, whereas the intensity ratios increased from 0.23 to 0.28 and 0.39 after 10 cycles and 100 cycles, respectively. This indicated that the protective layer guides the zinc crystal to epitaxial growth along the (002) direction due to the similar lattice between MXene and (002) plane of Zn.^[^ ^24^, ^25^ ^]^ Conversely, a sharp diffraction peak at around 8.0° in the bare zinc anode after 100 cycles appears (Figure S20, Supporting Information), assigning to the (001) plane of irreversible byproducts (Zn_4_(SO_4_)_4_(OH)_6_ 5H_2_O, PDF#39‐0688), which further support the severe corrosion reactions.^[^ ^4^ ^]^ In addition, the XPS results in Figure 4i reveal that there is no obvious difference in the S 2p spectra of zinc anode with the LM protective layer after 10 cycles and 100 cycles with a mere trace of ZnS presence, demonstrating corrosion‐free zinc plating/stripping.

**Figure 5:**
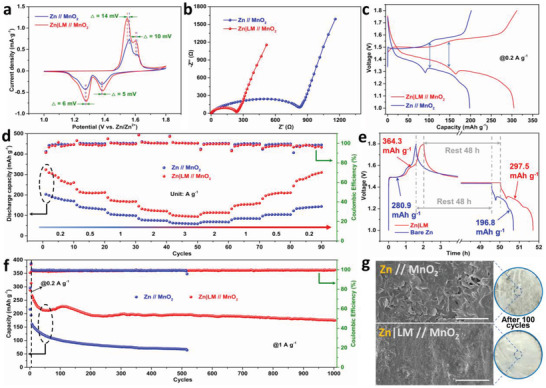
Electrochemical performance of full cells. a) CV curves at a scan rate of 0.5 mV s^−1^ (second cycle); b) Nyquist plots of Zn//MnO_2_ cells and Zn|LM//MnO_2_ cells. c) Galvanostatic charge/discharge profiles of full cells with/without the LM layer at a current density of 0.2 A g^−1^. d) Rate performance and coulombic efficiencies of the Zn//MnO_2_ cells and Zn|LM//MnO_2_ cells. e) Voltage‐time plots of full cells at 0.2 A g^−1^ (fully charged to 1.8 V, rest for 48 h, then fully discharging). f) Cycling performance of full cells with/without the LM layer at 1 A g^−1^. g) SEM surface morphologies of zinc anodes after 100 cycles. Scale bar: 10 µm.

To demonstrate the practical application of bifunctional LM layer, Zn//MnO_2_ full cells with/without the LM protective layer are assembled using the MnO_2_ cathode and the aqueous electrolyte (2 m ZnSO_4_ and 0.5 m MnSO_4_). As shown in Figures S21 and S22, Supporting Information, the synthesized MnO_2_ presents a nanorod‐like morphology with a tetragonal *α*‐MnO_2_ crystal structure (JCPDS 01‐072‐1982).^[^ ^26^ ^]^ The cyclic voltammetry (CV) profiles were first collected at a scan rate of 0.5 mV s^−1^. As shown in Figure S23, Supporting Information, both Zn//MnO_2_ and Zn|LM//MnO_2_ full cells exhibit similar shapes of redox peaks in the first three cycles, implying LM layer doesn't participate in the redox reactions.^[^ ^27^ ^]^ Besides, the Zn|LM//MnO_2_ cell displayed relatively higher reduction potential and lower oxidation potential than those of the Zn//MnO_2_ cell (**Figure** **5**a), suggesting the lower overpotential and higher reversibility induced by the LM protective layer.^[^ ^24^ ^]^ The results were further verified by the charge transfer resistance (*R*_ct_) obtained from the EIS curves. From the equivalent circuit in Figure S24, Supporting Information, and the Nyquist plots shown in Figure 5b, Zn|LM//MnO_2_ cell exhibits a much lower *R*_ct_ (242 Ω) than that of the Zn//MnO_2_ cell (836 Ω). Benefited from these merits, the Zn|LM//MnO_2_ cells deliver an initial discharge capacity of 304.9 mAh g^−1^ at a current density of 0.2 A g^−1^ (Figure 5c), which is much higher than that of the cells with the bare Zn anode (198.1 mAh g^−1^). As illustrated in Figure S25, Supporting Information, the Zn|LM//MnO_2_ cells maintain a high reversible capacity of 325.1 mAh g^−1^ with a CE of 99.6% after 100 cycles at 0.2 A g^−1^, much higher than those of Zn//MnO_2_ cells (188.8 mAh g^−1^ after 100 cycles), demonstrating high capacity and remarkable reversibility. The rate capability of Zn//MnO_2_ full cells with/without the LM‐protected layer was also compared (Figure 5d). With the current densities increased from 0.2 to 3 A g^−1^, the Zn|LM//MnO_2_ cells deliver an average capacity of 282.7, 212.9, 171.2, 126.8, and 98.2 mAh g^−1^ at each current density. When the current density returns to 0.2 A g^−1^, the Zn|LM//MnO_2_ cells maintain a high capacity of 285.8 mAh g^−1^. In contrast, the Zn//MnO_2_ cell without LM layer exhibits a lower average capacity of 183.0, 136.6, 102.8, 76.7, and 61.2 mAh g^−1^ at various current densities of 0.2, 0.5, 1, 2, and 3 A g^−1^, respectively. When the current density returns to 0.2 A g^−1^, Zn//MnO_2_ cells show a specific capacity of 137.3 mAh g^−1^, with a significant capacity loss of 25%. These results demonstrate that the designed LM protective layer effectively increases the specific capacity, improves cycling stability, and reduces resistance.^[^ ^28^ ^]^ Furthermore, to evaluate the effect of the LM layer on parasitic reactions of zinc anode, the discharge capacities of the fully charged cells (rest for 48 h) were measured (Figure 5e). Approximately 81.7% of the original capacity was retained in the Zn|LM//MnO_2_ cell, exceeding 70.1% in the Zn//MnO_2_ cell, implying the effective suppression of the side‐reactions at high voltage by the LM layer. In addition, the full cell with the LM protective layer can work stably for over 1000 cycles at a current density of 1 A g^−1^ (Figure 5f). The Zn|LM//MnO_2_ cells deliver a discharge capacity of 175.4 mAh g^−1^ with 90.0% retention of its average capacity after 1000 cycles, which is higher than those of the full cell without an LM layer (64.2 mAh g^−1^, 73.9% over 500 cycles) and other previous reported Zn‐MnO_2_ batteries (Table S1, Supporting Information). Considering the possible difference between Zn‐MnO2 full cell and Zn‐Zn symmetrical battery, the ex situ image of zinc plate is also compared. The dense and smooth surface of the zinc anode is protected with the LM layer after 100 cycles at 1 A g^−1^. In comparison, the bare zinc anode shows a rough surface with dendrites after 100 cycles (Figure 5g). These results indicate that the LM protective layer can inhibit the side‐reaction of zinc anode and improve its durability.

## 3 Conclusion

In summary, we implemented the synergic effect of mechanical induced dendrite suppression and zinc gating layer to enable dendrite‐suppressed and corrosion‐free zinc anode for long‐cycle‐life batteries with the LM layer. This protective layer not only inhibits the corrosion reaction by restricting the free water/anions molecules from the electrode/electrolyte interface through zinc gating, but also displays the high strength of the layer with low porosity to suppress the dendrite formation, shifting the diffusion limited dendrite formation to mechanical limited dendrite formation. Moreover, MXene with a similar lattice parameter with 002 plane of Zn, guides the zinc dendrite growth in the lateral direction through epitaxial growth. As a result, the zinc symmetric cells with the LM layer exhibit a longer cycling lifespan of 788 h and a lower polarization voltage of 35 mV than those in bare zinc symmetric cells (219 h, 194 mV). In addition, the Zn|LM//Cu asymmetric cells deliver a high CE of 98.9% and a low overpotential of 43.1 mV for over 200 cycles at 1 mA cm^−2^. Furthermore, the full Zn|LM//MnO_2_ cells achieve a higher capacity of 175.4 mAh g^−1^ and a long lifetime of over 1000 cycles at 1 A g^−1^, which is remarkably higher than those of the Zn//MnO_2_ full cell (64.2 mAh g^−1^, 73.9% over 500 cycles). It is believed that our strategy of application of the synergic effect of diffusion limited dendrite suppression and zinc gating function would provide further insight in developing other metal ion batteries for large‐scale application.

## 4 Experimental Section

### Synthesis of Lignin‐Containing Cellulose Nanofibers‐MXene Layer

LCNFs were rapidly extracted from sugarcane bagasse by microwave‐assisted deep eutectic solvents (DES) followed by ultrasonic treatment.^[^ ^17^ ^]^ The DES was prepared by the combination of choline chloride (ChCl) and lactic acid (LA) with a molar ratio of 1:10. The DES was utilized to pretreat the dried bagasse in a microwave reactor (LC‐JY99‐IIDN, Lichen Instrument Technology Co., LTD, Shanghai, China) to partially remove lignin. The microwave reaction time, temperature, and output power were set as 30 min, 120 °C, and 300 W, respectively. The obtained lignocellulose was dispersed into an aqueous solution with a concentration of 0.5 wt.%, and then sonicated by an ultrasonic processor (MCR‐3, Beilun Instrument Equipment Co., LTD, Shanghai, China) with a power of 1500 W for 30 min to produce an LCNF suspension. Ti_3_C_2_T_x_ MXene nanosheets were prepared according to the previous study.^[^ ^29^ ^]^ The as‐obtained MXene powder was dispersed in deionized water to form a suspension with a concentration of 5 mg mL^−1^. The LCNF suspension (6 g) was mixed with the MXene suspension (6 mL) with the assistance of ultrasonication for 1 h, followed by stirring for 24 h. Then, the LCNF‐MXene membrane was obtained by vacuum filtration of the mixture and drying at ambient temperature for 3 days. This membrane was designated as LM (LCNF‐MXene) layer.

### Synthesis of MnO2 Nanorods

MnO_2_ nanorods were prepared through a facile hydrothermal method.^[^ ^30^ ^]^ In brief, 0.1 m KMnO_4_ (30 mL) was added to 0.6 m MnSO_4_ H_2_O (30 mL). After stirring for 30 min at 25 °C, the mixture was transferred into a Teflon‐lined autoclave (100 mL) and kept at 140 °C for 12 h. Afterwards, the obtained product was washed by centrifugation with a mixture of water and alcohol, and then dried at 60 °C overnight.

### Characterizations

The chemical structure of samples was studied by an FTIR (VERTEX 80 V, Bruker Optics Inc., Ettlingen, Germany) with the attenuated total reflectance (ATR) mode, an X‐ray diffraction spectrometer (XRD, Ultima IV, Rigaku Cor., Tokyo, Japan) with Cu K*α* radiation at 40 kV and 30 mA, and a ThermoFisher Raman spectrometer (DXR532, Waltham, MA, USA) with a laser wavelength of 532 nm. The rheological properties of various film‐forming suspensions were measured using a rheometer (MARS60, Thermo Fisher Scientific Inc., Waltham, MA, USA) with a 20 mm diameter geometry and a 1° cone angle. The contents of elements and sulfate ions were analyzed by inductively coupled plasma mass spectrometer (ICP‐MS, NeXion 300X, PerkinElmer Inc., MA, USA) and ion chromatograph (ICS900, DIONEX Inc., Sunnyvale, CA, USA). The morphologies of samples were investigated by a field emission scanning electron microscopy (FESEM, Regulus 8100, Hitachi Co., Ltd., Tokyo, Japan) with an energy dispersive X‐ray spectroscopy (EDS) and a TEM (JEM‐1400, JEOL Ltd., Tokyo, Japan). The surface compositions and morphologies of zinc anodes were analyzed through an AXIS UltraDLD XPS (Shimadzu Cor., Berlin, Germany). In addition, a Bruker atomic force microscope (AFM, Dimension Edge, Tokyo, Japan) was employed to observe the surface and section morphologies of zinc anodes.

### Electrochemical Measurements

The Zn//Zn symmetric cells were assembled in CR2032 coin cells with two pieces of commercial Zn foil (50 µm in thickness) or modified zinc foil, glass fiber, and 2 m ZnSO_4_ as electrodes, separator, and electrolyte, respectively. In addition, the Zn//Cu asymmetric cells were prepared by replacing the Zn foil in the cathode side with Cu foil and the LM layer was placed on the top of Cu foil. The Zn//MnO_2_ batteries were assembled by CR2032 coin cells with MnO_2_ nanorods (mass loading: 1.0 mg cm^−2^), Whatman GF/A glass microfiber filter, and LM‐Zn layer as cathode, separator, and anode, respectively. 2 m ZnSO_4_ and 2 m ZnSO_4_ + 0.5 m MnSO_4_ aqueous solutions were employed as electrolytes. The MnO_2_ cathode was prepared through a mixture of 60% active materials, 30% acetylene black, and 10% PTFE binder. Galvanostatic discharge‐charge profiles were collected at ambient temperature by LAND‐CT3001A (Wuhan, China). CV, electrochemical impedance spectroscopy (EIS), and linear sweep voltammetry (LSV) measurements were conducted on a CHI760E electrochemical workstation (CH Instrument Inc., Shanghai, China). The EIS spectra were tested over the frequency range of 10^5^ to 10^−2^ Hz with a potential amplitude of 5 mV. The ex situ zinc dendrite growth was observed by an electrochemical workstation and a polarizing microscope equipped with a digital camera.

### Theoretical Calculations

The first‐principles calculations were carried out with density functional theory (DFT) to study the adsorption energy on LCNFs, MXene nanosheets, and the designed LM layer. For the DFT calculation, the Perdew–Burke–Ernzerhof (PBE) generalized gradient approximation (GGA) was employed as the exchange‐correlation functionals. The convergence tolerance of geometry optimization was set to 1) 1.0 × 10^−4^ eV atom^−1^ for energy, 2) 2.0 × 10^−2^ eV Å^−1^ for maximum force, and 3) 1.0 × 10^−3^ Å for maximum displacement. The planewave basis cutoff energy of 520 eV was used for the self‐consistent field method, while the 1×1×1 k point samplings were used within the Γ center Monkhorst–Pack scheme. For the van der Walls forces, the DFT‐D3 method of Grimme was used to get an accurate description of this force. I*β* cellulose structure was used for the calculations. The adsorption energy can be defined as: *E*_ad_ = *E*_tot_ − *E*_sub_ − *E*_mol_, where *E*_ad_, *E*_tot_, *E*_sub_, and *E*_mol_ denote the adsorption energy, the total energy of adsorption models, the energy of the matrix such as MXene nanosheets or cellulose, and the energy of the adsorption molecules such as H_2_O, Zn^2+^, and [Zn(H_2_O)_6_]^2+^, respectively.

## Conflict of Interest

The authors declare no conflict of interest.

## Supporting information

Supporting Information

## Data Availability

The data that support the findings of this study are available from the corresponding author upon reasonable request.
