# Supplementary material for: Synergic Effect of Dendrite‐Free and Zinc Gating in Lignin‐Containing Cellulose Nanofibers‐MXene Layer Enabling Long‐Cycle‐Life Zinc Metal Batteries
Source: Adv Sci (Weinh). 2022 Jul 7;9(25):2202380. doi: 10.1002/advs.202202380 (PMC9443465; doi:10.1002/advs.202202380)
Supplement: Supplementary file 1 — Supporting Information [file ADVS-9-2202380-s001.pdf]

ADVANCED SCIENCE
Open Access

# Supporting Information

for *Adv. Sci.*, DOI 10.1002/advs.202202380

Synergic Effect of Dendrite-Free and Zinc Gating in Lignin-Containing Cellulose Nanofibers-MXene Layer Enabling Long-Cycle-Life Zinc Metal Batteries

*Chaozheng Liu, Zhenglin Li, Xiaoman Zhang*, Wangwang Xu, Weimin Chen, Kangning Zhao*, Yao Wang, Shu Hong, Qinglin Wu, Mei-Chun Li and Changtong Mei**

# Supporting Information

**Synergic Effect of Dendrite-Free and Zinc Gating in Lignin-Containing Cellulose Nanofibers-MXene Layer Enabling Long-cycle-life Zinc Metal Batteries**

*Chaozheng Liu, Zhenglin Li, Xiaoman Zhang,* Wangwang Xu, Weimin Chen, Kangning Zhao,* Yao Wang, Shu Hong, Qinglin Wu, Mei-Chun Li and Changtong Mei**

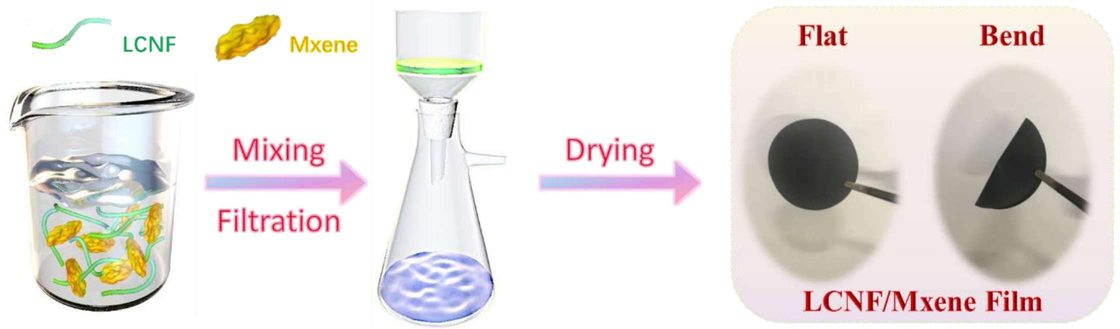


**Figure S1.** Preparation diagram and digital images of LM membrane.

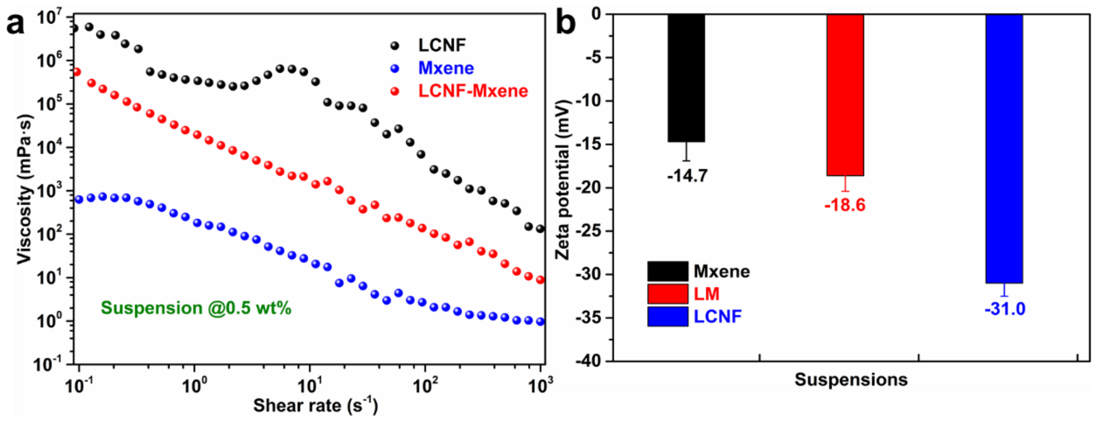


**Figure S2.** Rheological properties (a) and zeta potentials (b) of all three film-forming suspensions at a concentration of 0.5 wt%.

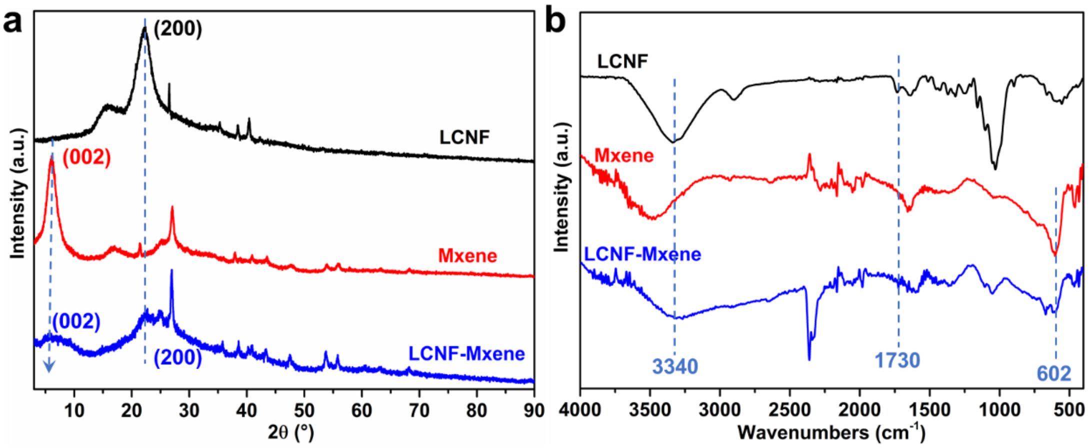


**Figure S3.** XRD patterns (a) and FTIR spectra (b) of LCNFs, Mxene and LM films.

As depicted in Figure S3a, the XRD patterns of the LCNFs film, Mxene film and LM film are investigated. The typical diffraction peak (200) at 2θ=22.4° of the LCNF assigned to the crystal plane of cellulose I crystalline structure is also detected in the pattern of the LM film. [1] Compared with the Mxene film, the diffraction peak of the (002) plane in the LM becomes weak and shifts to a lower position, respectively, which is due to the shielding effect of LCNFs and the intercalation of the LCNF nanofibers into the interlayer spaces between Mxene flakes. [2] In addition, the (002) peak shifts from 2θ=6.1° to 5.5°, indicating that the d-spacing increases from ~14.5 Å for the Mxene film to approximately 16.1 Å for the LM film, further confirming the successful intercalation of LCNFs into Mxene flakes. The FTIR spectra of the LCNFs film, Mxene film and LM film are shown in Figure S3b. Two characteristic bands of the LCNF at 3340 $cm^{-1}$ (assigned to the intramolecular hydroxyl groups of 3-OH...O-5 in cellulose) and 1730 $cm^{-1}$ (arisen from the ester and protonated carboxylic acid in lignin and hemicellulose), as well as the Mxene typical bands at 602 $cm^{-1}$ corresponded to the deformation vibration of the Ti-O bond are observed in the FTIR spectrum of the LM film. [3] These experimental results demonstrate that both LCNFs and Mxene can be connected to each other well, mainly due to the extensive interactions of hydrogen bond.

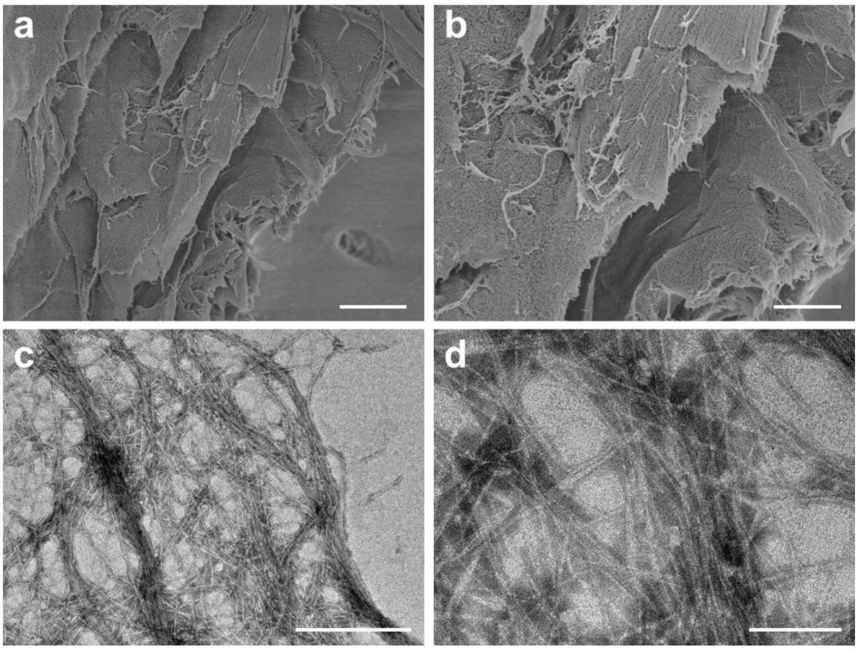


**Figure S4.** Morphologies of LCNFs. a, b) SEM images of LCNFs; c, d) TEM images of LCNFs. Scale bar: (a) 2 µm; (b) 1 µm; (c) 500 nm; (d) 100 nm.

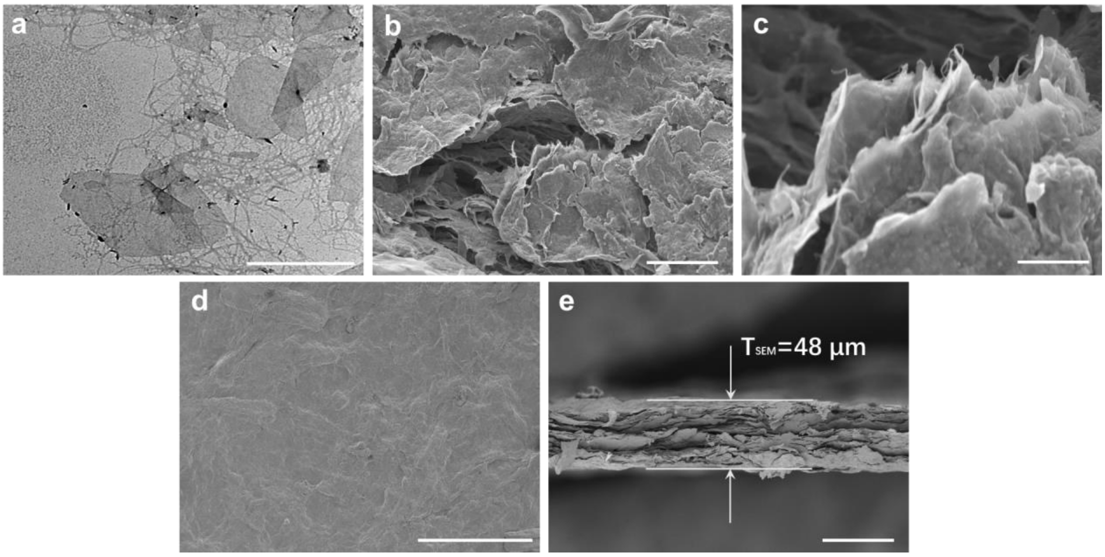


**Figure S5.** TEM morphology of LCNF-MXene suspension, and section and surface morphologies of LM film. Scale bar: (a) 1 µm; (b) 5 µm; (c) 1 µm; (d) 100 µm; (e) 50 µm.

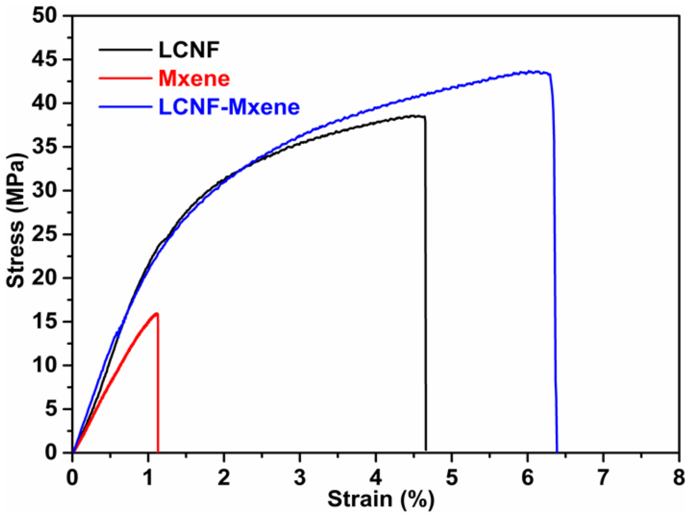


**Figure S6.** Mechanical properties of LCNF, LM and Mxene films.

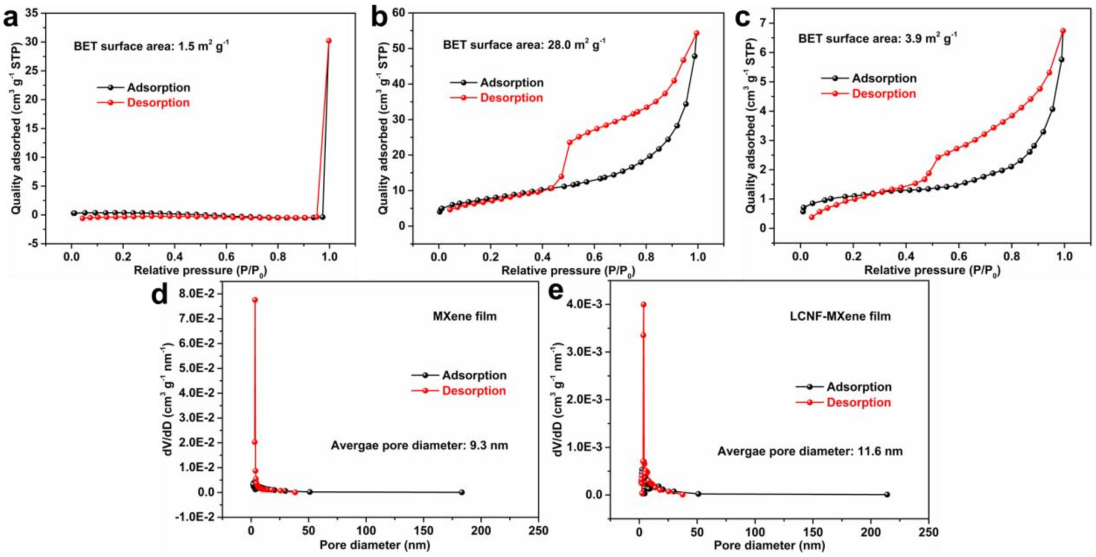


**Figure S7.** Nitrogen adsorption/desorption isotherms (a-c) and Barrent-Joyner-Halenda (BJH) pore size distributions (d, e) of LCNF, LM and Mxene films.

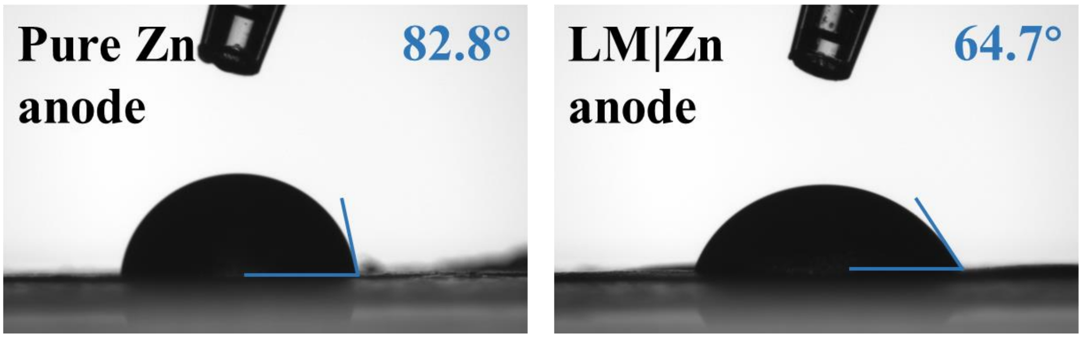

**Figure S8.** Contact angles of electrolyte on pure Zn anode and LM|Zn anode.

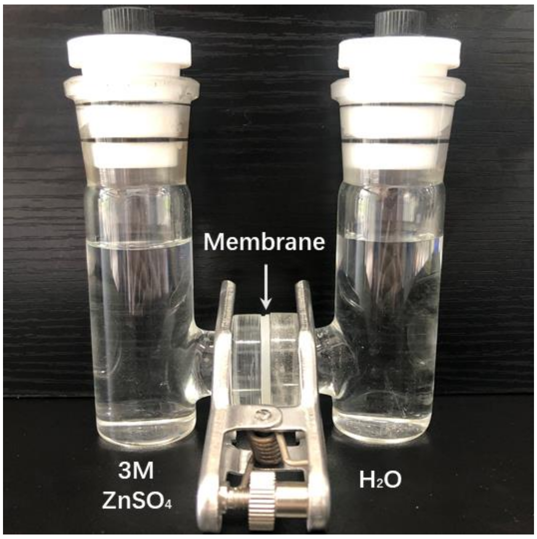


**Figure S9.** H-cells for $ZnSO_4$ permeability of LM film.

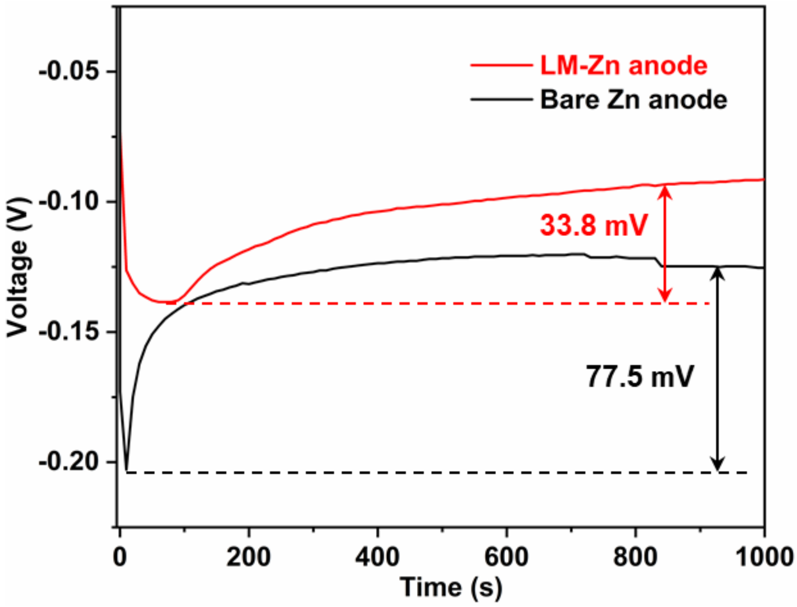


**Figure S10.** Voltage-time profiles of zinc anodes after Zn deposition for 1000 s at 5 mA $cm^{-2}$.

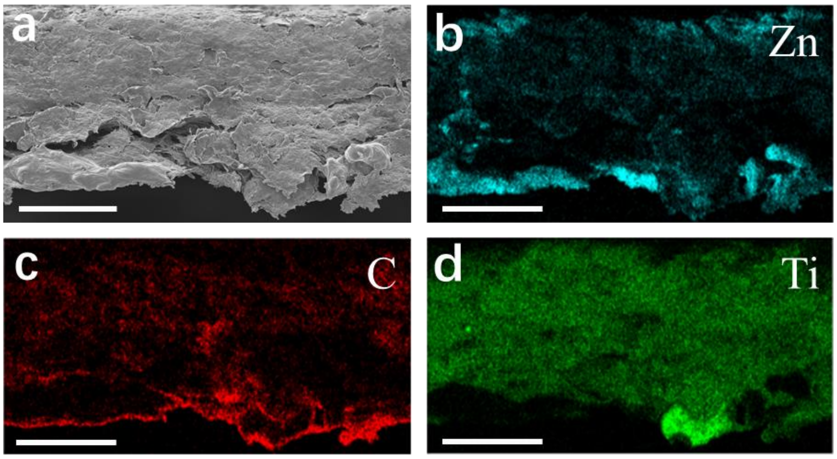


**Figure S11.** SEM (a) and the corresponding EDS mapping (b-d) images of LM layer after 1000 s deposition. Scale bar: 20 µm.

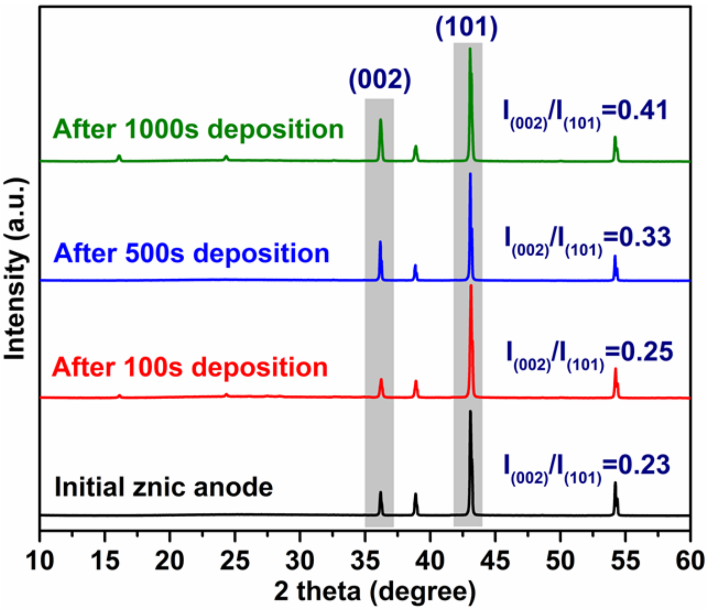


**Figure S12.** XRD patterns of zinc anodes protected with the LM layer after Zn deposition.

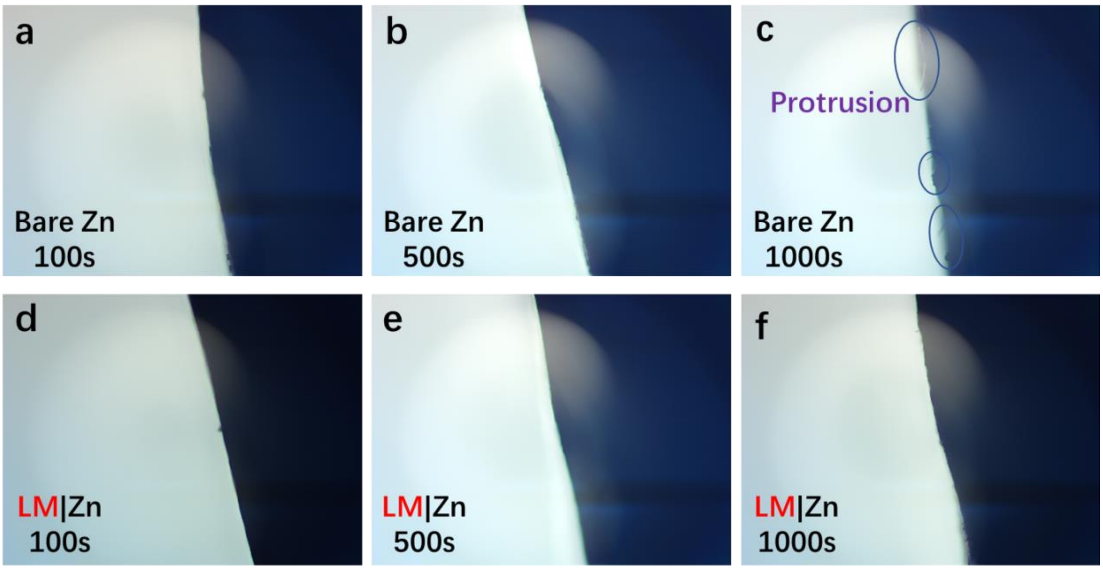


**Figure S13.** Optical visualization observations of Zn deposition of bare Zn anode (a-c) and LM-Zn anode (d-e).

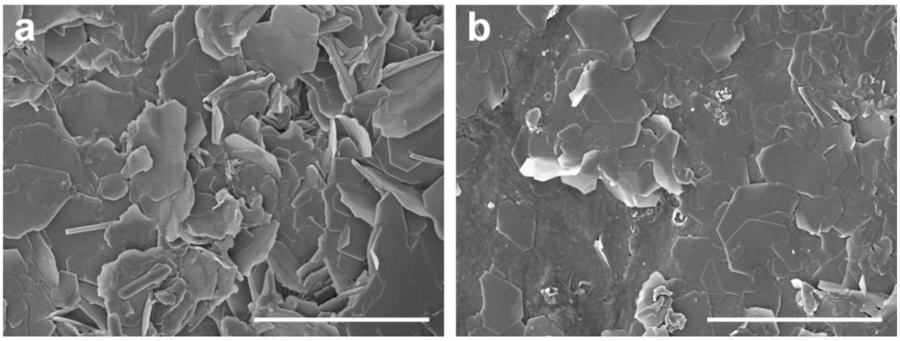


**Figure S14.** SEM images of bare zinc anode (a) and zinc anode protected with LM layer (b) at a current density of 5 mA·cm$^{-2}$ after deposition for 1000 s. Scale bar: 5 µm.

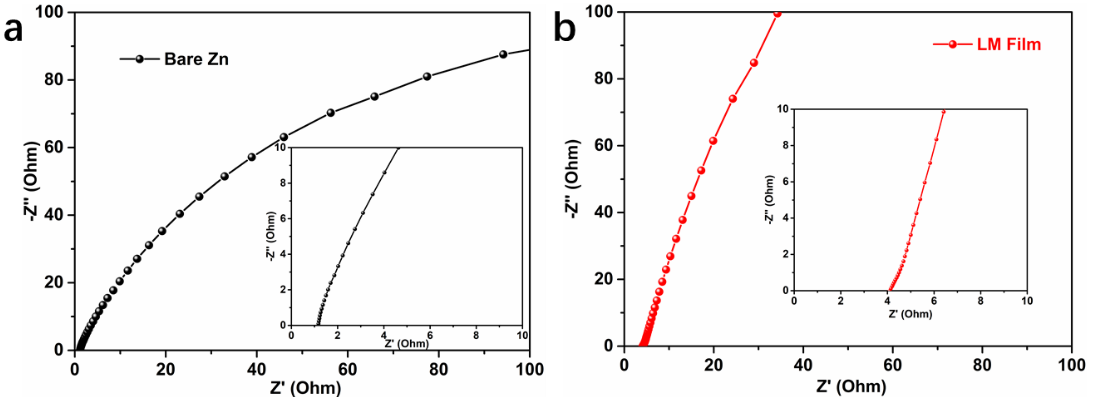

**Figure S15.** The EIS curves of bare zinc foil (a) and LM film (b).

The electrical conductivity (σ) was calculated through the following equation: σ=L/(R·S). L represents the thickness of samples (0.5 mm for bare Zn foil; 48 μm for LM film), R represents the resistance according to EIS curves (1.173 Ω for bare Zn foil; 4.147 Ω for LM film), and S represents the area of sample (4 $cm^2$). Therefore, the conductivity values of bare zinc foil and LM film are $1.07 \times 10^{-2}$ $S \cdot cm^{-1}$ and $2.89 \times 10^{-4}$ $S \cdot cm^{-1}$, respectively.

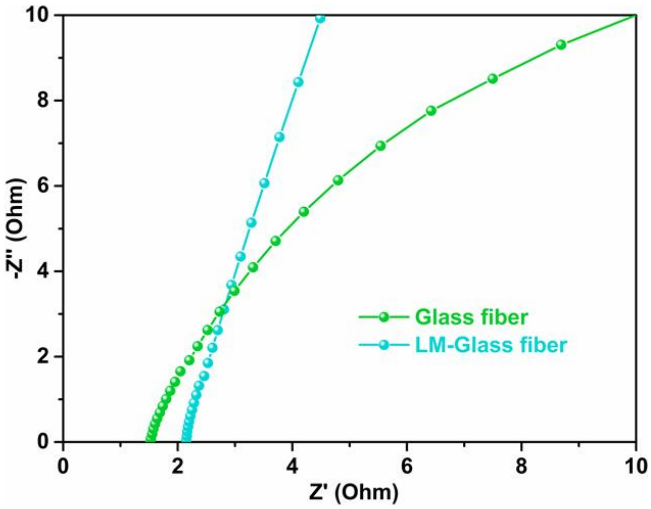


**Figure S16.** The EIS measurements of (a) Ti/Ti cell with glass fiber (GF) as separator, (b) Ti/Ti cell with LM-GF as separator.

The ionic conductivity (IC) of the separator was calculated through the following equation: [4] IC=d/AR. Where d (cm), A ($cm^2$) and R (Ω) are the thickness of the separator, the contact area, and the internal resistance (i.e., the intercept of the curve on X-axis in EIS curves), respectively. The resistance of GF and LM-GF separators are 1.532 and 2.148 Ω, respectively. For the LM layer, its resistance is 0.616 Ω ($\approx R_{LM\text{-}GF} - R_{GF}$). Therefore, the calculated IC of the LM layer is $1.55 \times 10^{-2}$ $S \cdot cm^{-1}$.

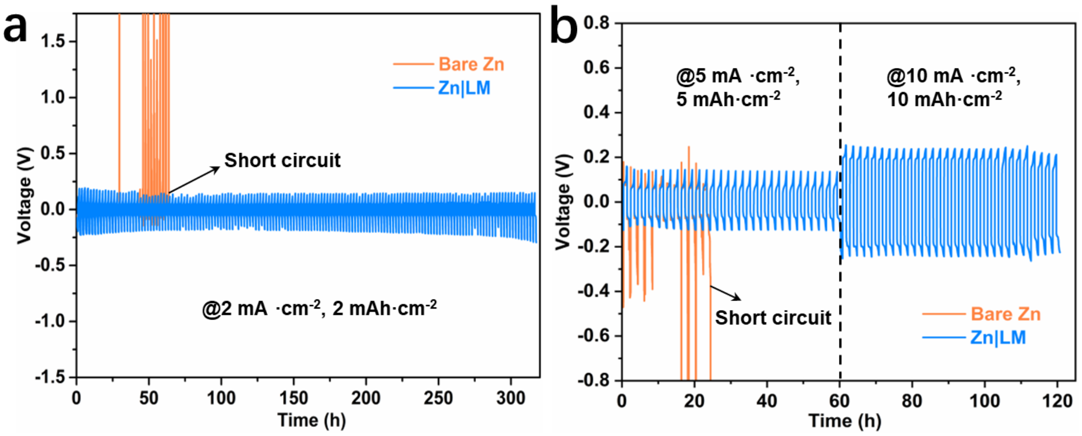


**Figure S17.** Galvanostatic cycling performance of zinc symmetric cells with/without the LM layer at different current densities and area capacities (a, 2 mA·cm$^{-2}$ with 2 mAh·cm$^{-2}$; b, 5 mA·cm$^{-2}$ with 5 mAh·cm$^{-2}$ and 10 mA·cm$^{-2}$ with 10 mAh·cm$^{-2}$).

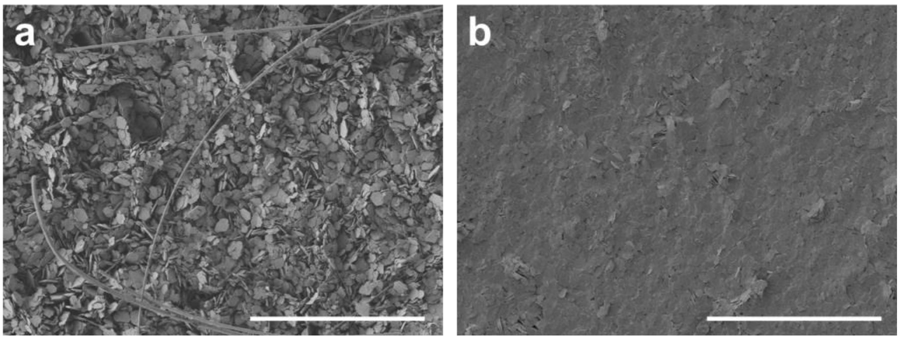


**Figure S18.** SEM images of bare zinc anode (a) and zinc anode with the LM protective layer (b) after 100 cycles under 1 mA·cm$^{-2}$ and 1 mAh·cm$^{-2}$. Scale bar: 100 μm.

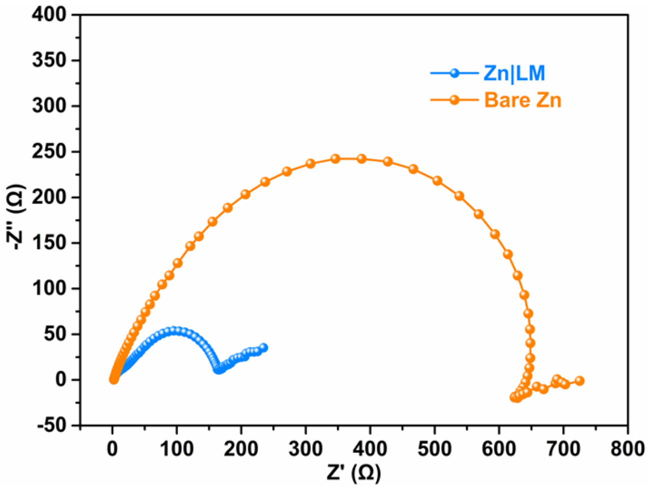

**Figure S19.** EIS curves of bare Zn//Zn cell and Zn|LM//Zn//LM cell after 100 cycles under 1 mA·cm$^{-2}$ and 1 mAh·cm$^{-2}$.

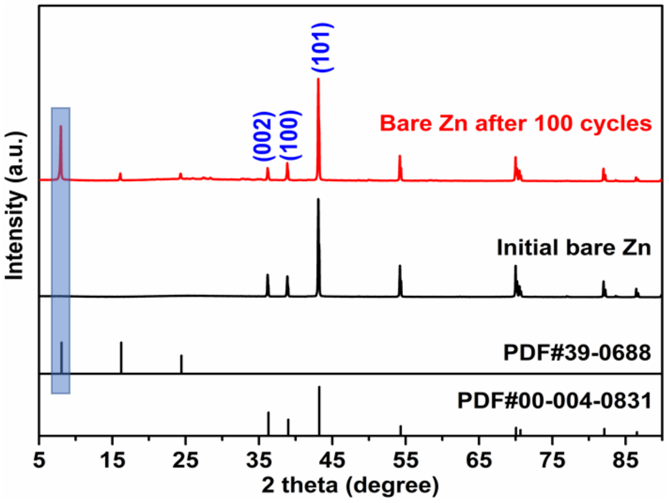


**Figure S20.** XRD patterns of the bare zinc anode before and after cycling at a current density of 1 mA·cm$^{-2}$ with a capacity of 1 mAh·cm$^{-2}$.

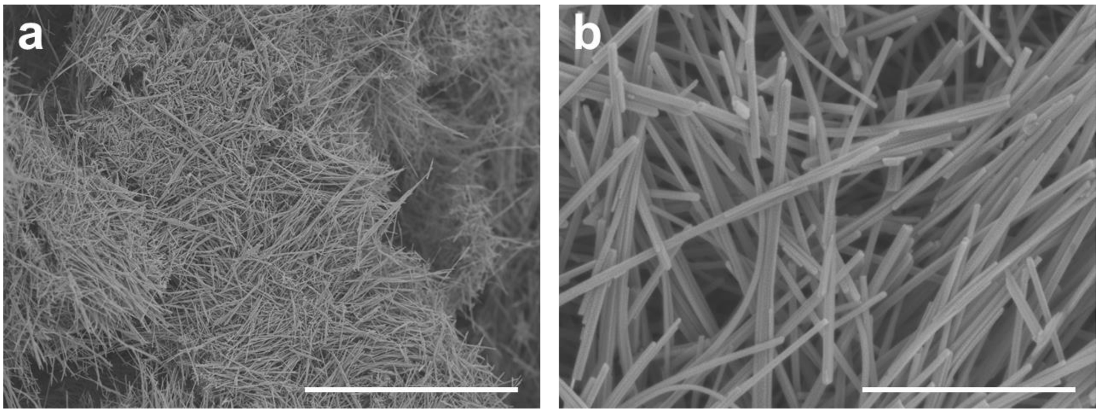


**Figure S21.** SEM images of $MnO_2$ cathode material. Scale bar: (a) 10 µm; (b) 1 µm.

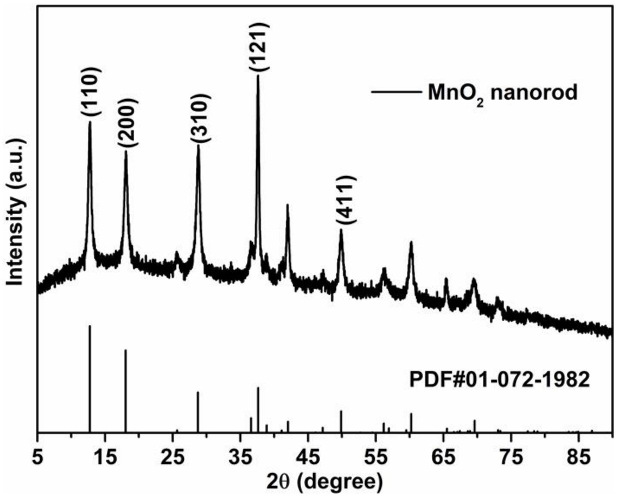


**Figure S22.** XRD pattern of the as-prepared $MnO_2$ nanorods

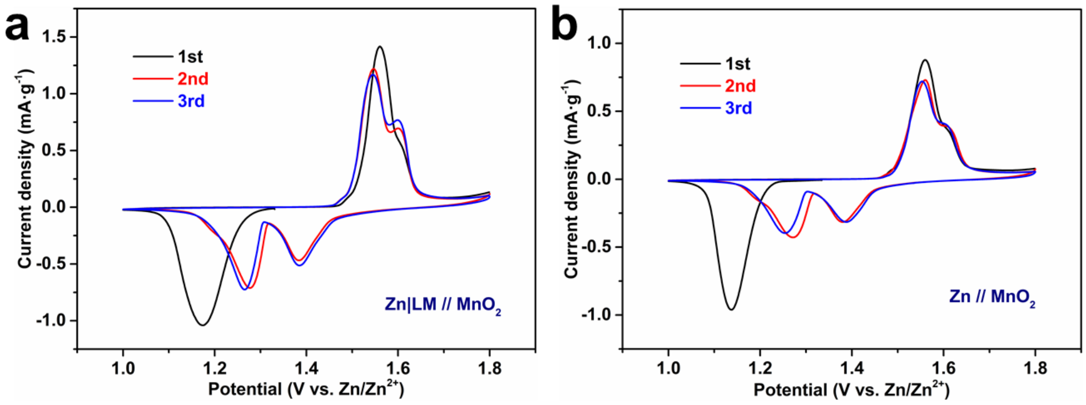


**Figure S23.** CV curves of Zn|LM // $MnO_2$ cell (a) and Zn // $MnO_2$ cell (b) at a scan rate of 0.5 $mV \cdot s^{-1}$.

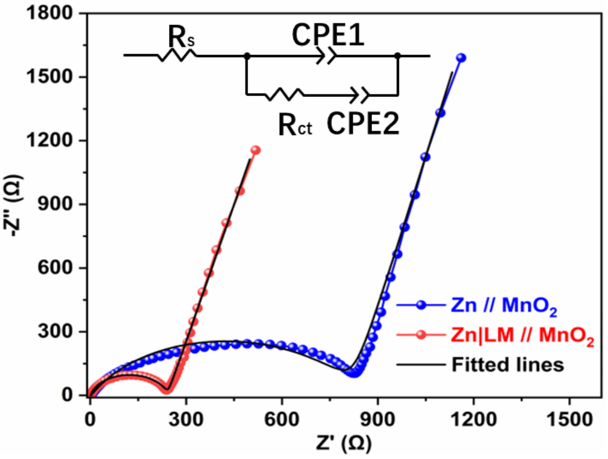

**Figure S24.** Nyquist plots and fitted lines of the full cells, and the inset is the equivalent circuit model of plot fitting.

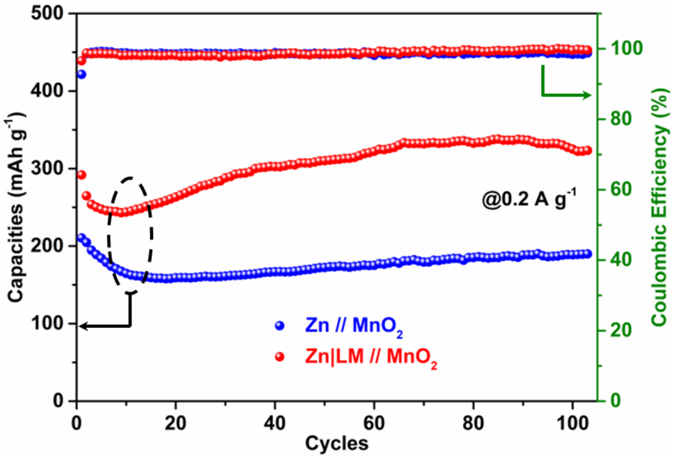


**Figure S25.** Cycling performance of full cells with/without the LM protective layer at 0.2 A·g$^{-1}$.

**Table S1.** Comparison of electrochemical performances of various $MnO_2$ cathodes.

| Cathode | Electrolyte | Capacity retention | Cycle life | References |
| --- | --- | --- | --- | --- |
| β-$MnO_2$ | 3 M $ZnSO_4$ + 0.1 M $MnSO_4$ | 94 % retention at 0.5 A·g$^{-1}$ | 500 cycles | [5] |
| α-$MnO_2$ | 2 M $ZnSO_4$ + 0.2 M $MnSO_4$ | 67 % retention at 7 A·g$^{-1}$ | 800 cycles | [6] |
| α-$MnO_2$ | 1 M $ZnSO_4$ | 45 % retention at 83 mA·g$^{-1}$ | 75 cycles | [7] |
| β-$MnO_2$ | 1 M $ZnSO_4$ | 75 % retention at 0.2 A·g$^{-1}$ | 200 cycles | [8] |
| β-$MnO_2$ | 2 M $ZnSO_4$ + 0.1 M $MnSO_4$ | 88 % retention at 0.2 A·g$^{-1}$ | 200 cycles | [9] |
| β-$MnO_2$ | 3 M $ZnSO_4$ + 0.2 M $MnSO_4$ | 83 % retention at 4 C | 1000 cycles | [10] |
| α-$MnO_2$ | 2 M $ZnSO_4$ + 0.2 M $MnSO_4$ | 81% retention at 1 A·g$^{-1}$ | 500 cycles | [11] |

| | | | | |
|---|---|---|---|---|
| $MnO_2$-graphene | 2 M $ZnSO_4$ + 0.4 M $MnSO_4$ | 64 % retention at 20 $mA\cdot cm^{-2}$ | 300 cycles | [12] |
| $MnO_2$@PEDOT | 2 M $ZnCl_2$ + 0.4 M $MnSO_4$ | 84 % retention at 1.11 $A\cdot g^{-1}$ | 300 cycles | [13] |
| α-$MnO_2$ | 2 M $ZnSO_4$ + 0.5 M $MnSO_4$ | 90 % retention at 1 $A\cdot g^{-1}$ | 1000 cycles | This work |

## References

[1] C. Liu, M.-C. Li, W. Chen, R. Huang, S. Hong, Q. Wu, C. Mei, Carbohydrate Polymers 2020, 246, 116548; A. D. French, Cellulose 2014, 21, 885.

[2] L. Yang, J. Cui, L. Zhang, X. Xu, X. Chen, D. Sun, Advanced Functional Materials 2021, 2101378; W.-T. Cao, F.-F. Chen, Y.-J. Zhu, Y.-G. Zhang, Y.-Y. Jiang, M.-G. Ma, F. Chen, ACS nano 2018, 12, 4583.

[3] J. A. Sirviö, M. Visanko, Journal of Materials Chemistry A 2017, 5, 21828; Y. Yue, J. Han, G. Han, Q. Zhang, A. D. French, Q. Wu, Carbohydrate polymers 2015, 133, 438; Q. Peng, J. Guo, Q. Zhang, J. Xiang, B. Liu, A. Zhou, R. Liu, Y. Tian, Journal of the American Chemical Society 2014, 136, 4113.

[4] C. Liu, W. Xu, C. Mei, M. Li, W. Chen, S. Hong, W. Y. Kim, S. y. Lee, Q. Wu, Advanced Energy Materials 2021, 2003902.

[5] M. Han, J. Huang, S. Liang, L. Shan, X. Xie, Z. Yi, Y. Wang, S. Guo, J. Zhou, iScience 2020, 23, 100797.

[6] B. Wu, G. Zhang, M. Yan, T. Xiong, P. He, L. He, X. Xu, L. Mai, Small 2018, 14, 1703850.

[7] M. H. Alfaruqi, S. Islam, J. Gim, J. Song, S. Kim, D. T. Pham, J. Jo, Z. Xiu, V. Mathew, J. Kim, Chemical Physics Letters 2016, 650, 64.

[8] S. Islam, M. H. Alfaruqi, V. Mathew, J. Song, Sj. Kim, Sh. Kim, J. Jo, J. P. Baboo, D. T. Pham, D. Y. Putro, Y. K. Sun, J. Kim, Journal of Materials Chemistry A 2017, 5, 23299.

[9] L. Li, T. K. A. Hoang, J. Zhi, M. Han, S. Li, P. Chen, ACS Applied Materials & Interfaces 2020, 12, 12834.

[10] M. Liu, Q. Zhao, H. Liu, J. Yang, X. Chen, L. Yang, Y. Cui, W. Huang, W. Zhao, A. Song, Y. Wang, S. Ding, Y. Song, G. Qian, F. Pan, Nano Energy 2019, 64, 103942.

[11] N. Zhang, S. Huang, Z. Yuan, J. Zhu, Z. Zhao, Z. Niu, Angewandte Chemie 2021, 133, 2897.

[12] C. Wang, Y. Zeng, X. Xiao, S. Wu, G. Zhong, K. Xu, Z. Wei, W. Su, X. Lu, Journal of Energy Chemistry 2020, 43, 182.

[13] Y. Zeng, X. Zhang, Y. Meng, M. Yu, J. Yi, Y. Wu, X. Lu, Y. Tong, Advanced Materials 2017, 29, 1700274.
